# How do NHS organisations plan research capacity development? Strategies, strengths, and opportunities for improvement

**DOI:** 10.1186/s12913-018-2992-2

**Published:** 2018-03-22

**Authors:** Melanie Gee, Jo Cooke

**Affiliations:** 1 Faculty of Health and Wellbeing, Sheffield Hallam University, Montgomery House, 32 Collegiate Crescent, Collegiate Campus S10 2BP, Sheffield, UK; 2 NIHR CLAHRC Yorkshire and Humber, Sheffield Teaching Hospitals NHS Foundation Trust, Room D33, D Floor, Royal Hallamshire Hospital S10 2JF, Sheffield, UK

**Keywords:** Research capacity development, Organisational infrastructure, Research funding, Health systems research

## Abstract

Research that is integral into a ‘learning healthcare system’ can promote cost effective services and knowledge creation. As such, research is defined as a ‘core function’ in UK health service organisations, and is often planned through research and development (R&D) strategies that aim to promote research activity and research capacity development (RCD).

The discussion focuses around the content of ten R&D strategies for healthcare organisations in England and Scotland, with respect to RCD. These organisations were engaged with a research interest network called ACORN (Addressing Organisational Capacity to do Research Network) that included two Scottish Health Boards, four community and mental health trusts, two provincial district hospitals, and two teaching hospitals.

We undertook a thematic documentary analysis of the R&D strategies which identified 11 ‘core activities’ of RCD. The potential for building research capacity in these ‘core activities’ was established by reviewing them through the lens of a RCD framework.

Core activities aimed to ‘hard wire’ RCD into health organisations. They demonstrated a complex interplay between developing a strong internal organisational infrastructure, and supporting individual career planning and skills development, in turn enabled by organisational processes. They also included activities to build stronger inter-organisational relationships and networks. Practitioner, manager and patient involvement was a cross cutting theme. The potential to demonstrate progress was included in plans through monitoring activity across all RCD principles. Strategies were primarily aimed at research production rather than research use. Developing ‘actionable dissemination’ was poorly addressed in the strategies, and represents an area for improvement.

We describe strengths of RCD planning activities, and opportunities for improvement. We explore how national policy and research funders can influence health systems’ engagement in research.

**Electronic supplementary material:**

The online version of this article (10.1186/s12913-018-2992-2) contains supplementary material, which is available to authorized users.

## Background

### Research Capacity Development in Healthcare Systems

There is broad consensus that healthcare systems should integrate research in order to promote health, wealth and knowledge creation [1]. Faden et al. [2] suggest that it is both ethical and moral to support ‘learning healthcare systems’ that integrate research and healthcare practice through continuously studied, tested and improved services. Many authors support a ‘whole systems’ approach in order to strengthen a research culture, and increase research capacity within organisations and the workforce [3–5].

The current national policy in the UK calls for ‘NHS [National Health Service] and patient participation in research to improve outcomes and promote economic growth’ [6], recognizing the need for developing cost effective services, and developing links with industries to impact on the economy. The NHS Constitution [7] refers to ‘the promotion, conduct and use of research to improve the current and future health and care of the population’. There is therefore recognition across NHS trusts that research should become embedded into the organisation - it should be part of ‘core business’.

Healthcare systems need to invest and plan strategies and activities to develop research capacity. Research Capacity Development (RCD) has been defined as ‘a funded, dynamic intervention operationalized through a range of foci and levels to augment ability to carry out research or achieve objectives in the field of research over the long-term, with aspects of social change as an ultimate outcome’ [8]. The current NHS R&D policy ‘Best Research for Best Health’ [9] offers some opportunities for Trusts in shaping and resourcing these strategies. This policy instigated a National Institute of Health Research (NIHR), with an ambition to support research ‘reach’ into each NHS organisation. It aims to do this firstly, by funding a Clinical Research Network (CRN) whose function is to support research delivery in the NHS. Resource supports clinician time and recruitment in each organisation, and additional funds are linked to activity and efficiency of recruitment. Secondly, by developing a NIHR Faculty. This aims to identify, recognise and resource research active staff. It also offers funding opportunities for clinical academic careers through a series of ‘stepped’ fellowships. Thirdly, by commissioning research programmes that clinical academics could compete for, with the aim of providing benefit to patients. And finally, by creating systems to manage and support research and its outputs that are embedded in the NHS, including research ethics and governance systems.

As Condell and Begley [8] observe, some authors use RCD interchangeably with RCB (Research Capacity Building). In this paper we use the term RCD, as the term ‘development’ conveys activities around expanding and upgrading pre-existing capabilities in the organisation, rather than starting from scratch [10].

### Incorporating RCD into organisational planning: ACORN

A research interest network called ACORN (Addressing Capacity in Organisations to do Research Network) was developed as part of the capacity programme within a Collaboration and Leadership of Applied Health Research and Care in Yorkshire and Humber (CLAHRC YH), in the North of England. CLAHRCs are National Institute of Health Research (NIHR) funded collaborations, with a key objective to build health services research capacity. ACORN was developed to build a community of practice (CoP) as this approach has become increasingly influential within management research and practice to drive capacity development in these organisations [11]. CoP is described as ‘a vehicle for collective learning in a field of shared human endeavour, enhanced by mutual concerns, passions and regular group interactions’ [12]. An agreed aim at the outset in the development of the ACORN community was to share the research and development (R&D) strategies within the group, and this enabled a cross documentary review of strategies. This paper is an outcome of this review, which aims to look for common themes and explore joint learning for the ACORN group and others interested in building RCD at an organisational level.

At the time of this R&D strategies review, ACORN comprised ten NHS organisations, including two Scottish Health Boards and eight trusts from the north of England. It included four community and mental health trusts, two provincial district hospitals, and two teaching hospitals. All ACORN members had R&D strategies published before the group was developed, some covering the whole organisation, and others that focussed on nursing and Allied Health Professionals (AHPs) (see Table 1). All the strategy documents articulated overarching strategy aims (e.g. “to increase the volume and quality of applied research that leads to improvements in patient/client health and well-being and service delivery”; “to offer training opportunities for the public, service users and carers who have expressed an interest in active involvement in research”; “to maximised the use of research to support cost efficiencies”). They all incorporated implementational planning elements, to differing levels of detail: all described RCD activities that were being planned or undertaken in each organisation, and some additionally described milestones, measurements and key performance indicators associated with those activities. Our aim was to categorise the RCD activities, and identify ‘core activities’ by determining which ones were the most frequently described across this diverse range of organisations.

**Table 1:** Summary characteristics of ACORN organisational R&D strategies examined

Strategy ID	Type of trust	Period covered by strategy	Author of strategy	Focus of strategy
1	District hospital	2014–2018	Director of R&D	Whole trust
2	District hospital	2013–2018	Director of R&D	Whole trust
3	Teaching hospital	2015–2020	Not stated	Whole trust
4	Teaching hospital	2011–2015	Professional Services Research Executive	Professional Services Directorate
5	Community, and mental health, and learning disability	2010–2015 and 2014–2015 (PPI strategy)	Not stated	Whole trust
6	Community health	2012–2015	Chair, Chief Executive, Executive Medical Director, Executive Director of Quality	Whole trust
7	Mental health	2013–2016	Director of Research	Whole trust
8	Mental health and learning disability	2014–2017	Not stated	Whole trust
9	Scottish Health Board	2014–2019	Not stated	Nursing & Midwifery
10	Scottish Health Board	2011–2015	Not stated	Nursing, Midwifery & Allied Health Professionals

these principles are provided in Table 2

We also wanted to see how these activities compared to the evidence of what works in RCD. We did this by describing how they mapped against the six principles of an adapted version of Cooke’s evidence based framework [13]. The Cooke framework was developed through the blending of knowledge from analysis of the literature, R&D policy documents, and the experience of one Research and Development Support Unit in the UK. It has been further developed with a particular focus on healthcare organisations [14, 15]. The ACORN CoP have agreed to use it to review current activity, and plan further work. This documentary analysis is the first step in this process.

**Fig. 1:**
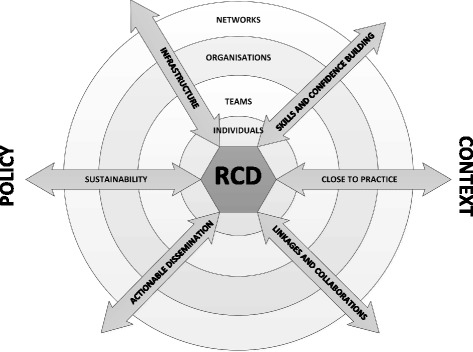
Adapted Cooke framework for RCD. (Adapted from Cooke’s evidence-based framework [13])

The adapted version of Cooke framework that we used is shown in Fig. 1. It contains the following principles, or ways of doing RCD: promoting actionable dissemination (DISS); developing research ‘close to practice’ (CTP); developing a support infrastructure (INF); supporting linkages and collaborations (LINKS); developing research skills and confidence in the health services workforce (SKILLS); and planning sustainability (SUS). Our definitions for.

The utility of the Cooke framework for RCD analysis and evaluation has been demonstrated [16–18], and this framework has been widely used in supporting RCD in a range of contexts [19, 20]. In undertaking this methodology we were able to capture core activities that may be transferable to other healthcare contexts, and by looking through the lens of the adapted framework we were able to determine the theoretical underpinning of how such activities can contribute to capacity building endeavour. Thus we ascertain our findings may be a useful starting point for discussion for others planning RCD in health services.

## Main Text

### Documentary analysis of the organisation R&D strategies

We performed a documentary analysis of the ten ACORN members’ R&D strategies, using NVivo 10 Software [21]. We only coded parts of the strategies describing ongoing or planned activities associated with RCD; some parts of the strategies, such as context-setting and information about wider organisational developments, were not coded. We used open coding to label the RCD activities described in the documents. Coding was carried out by one reviewer (MG) but areas of doubt or ambiguity were discussed with the second reviewer (JC) who also double-coded a sample from one strategy as part of the code-checking process. Where it was not immediately obvious how to code any section of text, node-linked memos were created to capture the decision-making, and node definitions were developed and refined as appropriate. Having coded all the strategies, we interrogated NVivo to check for coding consistency between the strategies, and refined and consolidated the activity nodes (e.g. where different labels were applied to essentially the same sort of activity). The final coding tree for the activity codes is provided in Additional File 1.

We identified those RCD activities which were evident in at least 7/10 strategy documents, from our coding in NVivo. These ‘core activities’ are listed on the left hand side of Table 3. We then viewed the data pertaining to each of these core activities through the lens of Cooke’s adapted framework to ascertain which RCD principles from the framework were being used to address research capacity. This was enabled through our shared understanding of the scope of each principle, and collaborative production of the principle definitions presented in Table 2. The mapping between core activities and RCD principles is shown in Table 3.

**Table 2:** The principles of RCD

Promoting actionable dissemination (DISS)	This principle relates to dissemination of research findings through a range of methods. It can include traditional scholarly methods, (publications, conference presentations) or other means including websites, multi-media or tools and techniques to support decision making in practice. Dissemination can be internal to the trust or external and ‘actionable’ implies some sort of impact - scholarly impact, and/or impact on practice (e.g. in policies, instruments, programmes of care, or factsheets). In the latter there are therefore links to the CTP principle.
Developing research ‘close to practice’ (CTP)	This principle relates to research being delivered or developed within health services, thus co-producing research with high level of relevance to practice or policy concerns. It can relate to research questions and priorities being set by or with practitioners and services, policy makers, and/or service users, using practice or experiential knowledge. It can also relate to relevant research being ‘impactful’ and becoming embedded into practitioners’ day-to-day activities.
Developing a support infrastructure (INF)	This principle relates to building additional resources, and/or processes into the trust’s organisational system to enable the smooth and effective running of research projects and for research capacity building.
Supporting linkages and collaborations (LINKS)	This principle relates to forming links, both internal and external, and on an organisational and individual level, to enhance RCD through knowledge exchange and collaboration. It can also relate to benefiting from resources and services beyond the trust.
Developing research skills and confidence in the health services workforce (SKILLS)	This principle relates to training and development opportunities to create a workforce with the skills and confidence they need to conduct research, apply for funding, lead on research projects, and for career progression opportunities.
Planning sustainability (SUS)	This principle relates to ensuring that the existing level of research capacity can be sustained, and ideally, grown. This principle therefore overlaps with INF, LINKS, and SKILLS, as many of the activities associated with these other principles will also contribute to sustainability.

**Table 3:** How core RCD activities map against RCD principles

		Principles from Cooke’s adapted framework					
		Actionable dissemination (DISS)	Close to practice (CTP)	Infrastructure (INF)	Linkages and collaborations (LINKS)	Skills and confidence building (SKILLS)	Sustainability (SUS)
Core activities described in the R&D strategies (present in at least 7/10 organisations)	Developing and sustaining research collaborations		✓	✓	✓	✓	✓
	Developing research priorities		✓	✓	✓		✓
	Academic dissemination	✓				✓	
	Evidence based practice and knowledge transfer		✓	✓			
	Hard wired into the organisation: making research core business		✓	✓		✓	
	Proactive and timely communication of research opportunities			✓			
	Patient and public involvement and engagement in research		✓	✓	✓	✓	
	Research governance support			✓	✓		
	Research education and learning		✓	✓	✓	✓	
	Setting targets and monitoring performance	✓	✓	✓	✓	✓	✓
	Internal investment: allocating resources to promote research capacity		✓	✓	✓	✓	✓

### Evidence for core RCD activities in the R&D strategies

We now provide a description of the core RCD activities we identified, indicating in parentheses which of the RCD principles apply.

#### Developing and sustaining research collaborations

Most strategies described activities relating to external research collaborations and developing research proposals and competitive bids, both of which relate to sustainability planning through increased externally-funded research activity (SUS).

Investment in nurturing external collaborations and networks (LINKS) was seen to increase the likelihood of gaining external funding. Links with academics working within universities (including the provision of joint academic posts), the NIHR CRN, and funded collaborations such as CLAHRC, were strongly supported. These links also provided opportunity for research training and development (see under ‘Research skills development’ below) (SKILLS). One strategy sought evidence of sustained clinical-academic dialogue through joint working groups and collaborative agreements. The NHS organisations recognised their unique position of increasing access to patient groups within research partnerships (see ‘Patient and public involvement and engagement below) (CTP).

Some strategies also described links with commercial partners, in particular with regard to priority setting and targets (see under ‘Developing research priorities’ and ‘Setting targets and monitoring performance’ below).

Research ‘business’ plans were often described, aimed at capturing funds through being a recruitment site for high quality nationally and commercially funded projects (called portfolio projects by the NIHR). Partnership with the NIHR CRN) was seen as important in this regard, as this network funds time for clinical researchers and research nurses to recruit patients to portfolio studies so as not to impinge on clinical budgets. Additionally, the CRN can contribute to research infrastructure by funding support for governance and ethics applications (LINKS and INF). This highlights how the national policy around research delivery has influenced local strategy and reach into NHS organisations.

Research funded through competitive tendering was seen to be a way of engaging with research of high quality and increasing the reputation of the trust. The influence and impact for conducting this type of research on clinical care was also seen as important (CTP). The NIHR as a funding body was prioritized by trusts, as such grants attracts additional ‘between’ grant funds to NHS organisations (SUS) for practitioners with an ambition and ability to do research. This is called Research Capability Funding (RCF) and is aimed at increasing research capacity in the NHS. Such grant income was therefore seen as doubly effective in funding activity and strengthening capacity, and thus was included as an element of the business case for R&D.

#### Developing research priorities

Most strategies highlighted the need to align research activity to wider organisational strategic objectives, business planning, quality strategies or audit activities (INF). Many emphasised the role of service users, carers and the public in priority setting to ensure that research was relevant and of benefit to patients (CTP). Mechanisms for Public and Patient Involvement (PPI) included links with patient and carer research groups. One trust aimed to develop a more systematic approach to eliciting patient views through asking about research priorities in patient experience surveys that were routinely undertaken for quality assurance in care provision. Another trust planned to run a development day/workshop to identify and prioritise research questions from service user, carer, and professional perspectives.

Research coproduction encompassing priority setting with wider partners was planned. This included coproduction with academic partners, private and voluntary sector organisations, and research networks (LINKS), and this was seen as a way of attracting further income (SUS). One strategy stated that ‘a focused approach, building research expertise and experience in a few key areas, is more likely to be successful in establishing local programmes of research that are nationally competitive than a ‘scattergun approach’.

#### Academic dissemination

Research dissemination featured in most strategies. Some explicitly referred to creating and implementing dissemination strategies (DIS). This predominantly focussed on academic dissemination in peer-reviewed publications in order to enhance individual and organisational research profiles. Many strategies planned activities to develop staff members’ academic writing skills (SKILLS) and engender a culture of support with an expectation to publish as an outcome. Publications were often cited as a key performance indicator for the impact of R&D strategies, along with the number of oral and poster conference presentations.

Fewer strategies prioritised *local* research dissemination (i.e. within the organisation and to affected patient groups). However, examples of internal publications were an e-journal/newsletter, and an R&D magazine. One trust planned to maintain an internal Publication Register to support appropriate dissemination, and another planned to ask researchers to write research summaries for internal dissemination. Internal research conferences and events to ‘showcase’ and celebrate activity were planned by several trusts. These, again, may be a mechanism for raising the organisation’s research profile within and outside the organisation.

#### Evidence-based practice and knowledge transfer

All the strategies referred to a desire to translate the trust’s research into local practice, with a resultant impact on patients and service transformation (CTP). There was a recognition that this was more likely to happen if the research has maximum local relevance: as described under ‘Developing research priorities’ above, several strategies described ongoing and planned working with key partners to ensure this.

Several strategies identified the need to build infrastructure to support evidence-based practice and knowledge transfer (INF). These included mechanisms for internal dissemination of research findings and identifying key staff to follow them up for service improvements (academic local research dissemination is discussed above under ‘Academic dissemination’); involving general managers in knowledge mobilisation programmes; and using community of practice groups.

#### ‘Hard-wiring’ research into the organisation

This activity was strongly associated with making research ‘core business’. All strategies included mechanisms to ensure research would become ‘hard-wired’ into the organisation, i.e. embedded in its day-to-day activity. These mechanisms fell into two broad categories: firstly, linking research planning to wider organisational and business planning processes; and secondly, providing an explicit expectation that individual members of staff would engage in research activities.

All strategies described links between RCD and organisational business planning (INF). Some described alignment with the overall strategic direction of the organisation, and in one, the research strategy objectives were explicitly mapped to corporate priority strategic objectives. For example, the research objective ‘To develop collaborative working with patients/public in research’ was mapped to the corporate objective ‘To work in partnership with service users, communities and stakeholders to deliver service solutions, particularly around integrated care and care closer to home’. At a local level, some strategies referred to linkages between the organisational research aims and smaller clinical units within them (CTP).

R&D strategies often highlighted internal policy links that offered opportunities for alignment and synergy, including intellectual property (IP) policy, staff training, and models for PPI (INF). Including patient’s views was embedded within the systems of several trusts to identify priorities (see ‘Patient and public involvement and engagement in research’ below).

A commonly cited philosophy was that research should be everyone’s business, from the top to the bottom of the organisation. For example some strategies planned to report research performance as a regular item on the trust Board agenda (INF). Many referred to plans for integrating an expectation of research activity into working practice through job descriptions for existing staff and in advertised posts, in job plans, and professional development pathways (INF, CTP). Providing protected time for research activities, either through job planning or ‘release’ of staff, was also recognised as important for developing research skills (SKILLS), in order to enable clinical staff members to ‘learn by doing’. Engaging clinical managers in the research agenda was also thought to be beneficial in order to support the workforce in performing research activities, and to build this into appraisal and performance management processes (SKILLS and INF).

#### Proactive and timely communication of research opportunities

Rapidly identifying, assessing, and communicating the relevant research opportunities to the right people were seen as important, requiring organisational infrastructure to support this (INF). Some trusts already used, or planned to use, dedicated research support staff to fulfil this role. Others worked in clinical-academic partnerships to do this. Trust websites, intranet sites or research share points were referred as mechanisms for disseminating opportunities in the future. One trust planned to send monthly reports directly to clinical staff at clinical speciality level.

#### Patient and public involvement and engagement in research

Most strategies supported PPI in research, motivated by improving the quality of care provided by the NHS through research (CTP). One strategy stated that ‘Involving patients and public in research can lead to more appropriate people centred care, improved health outcomes and sustainable solutions.’ Ongoing dialogue with patients was seen as an important function for NHS partners in academia partnerships (LINKS). Many trusts had identified funding to support PPI, through NIHR bodies (LINKS).

Some trusts had developed a directory of patient groups with specific conditions willing to support R&D functions, whilst others developed groups specifically to undertake research governance functions (INF), sometimes within wider research collaborations (LINKS). These groups received training and support (SKILLS) from NHS R&D departments (INF) to enable engagement in research development. PPI involvement is a requirement for many funding bodies, therefore many strategies aimed to budget for PPI in projects. One trust had a target that PPI should between 10 and 20% of the total costs of funding applications. Many trusts also aimed to involve patients in the dissemination of research findings in conferences, newsletters and films/videos.

Some planned PPI activity included increasing the general public’s awareness that research is part of core NHS business, for instance including statements to that effect in clinic appointment letters, or through creating patient ambassador roles (INF). Increased public awareness of projects that exist in trusts would, it was hoped, support patient recruitment, increase the quality of care, and also impact on R&D business case (funds follow recruitment numbers). One trust planned to survey patients and members of the public involved in research to find out their experiences and how they might be involved in the future in order to increase recruitments and participation.

Many trusts aimed to evaluate the impact of PPI. For example one trust intended to survey people involved in PPI activity in order to ‘ensure appropriate levels of recognition and reward for involvement are maintained’. Another intended to identify the number of projects that included PPI within them, and to describe this activity in detail.

#### Research governance support

The Department of Health has clear regulatory and legal requirements for the conduct of research in the NHS [22]. Existing or planned research governance and support offices were an important constituent in the strategies (INF), whether provided through trust-based research support offices, or by partner organisations (LINK). Such offices are able to navigate the system, reducing potential barriers to research participation and enabling research to progress, thus increasing research capacity.

#### Research education and learning

Most strategies included the planned provision of research skills training and development for research-active clinical staff (SKILLS), and in some cases also to other clinical staff, and managers. Two strategies planned to review training arrangements by research management groups (INF). Supporting skills around change management and innovation was recognised as a way of enabling learning through change and sharing that learning with others (CTP).

Planned training activities included workshops for generic research methods such as study design, accessing and appraising evidence, ethics, research governance, and writing for publication. Such training would be provided in-house (INF) or provided through academic networks such as the CLAHRC YH (LINKS). One strategy referred to commissioning bespoke training, and two planned to identify funding opportunities to support training.

A key theme was support for new and emerging researchers. This included mechanisms to identify clinicians with a research ambition, and to maintain a database of staff with research potential (INF). One larger trust offered an intensive ‘Research Boot Camp’ focussing on grant applications and publications for early career ‘clinical researchers, with associated mentoring and peer support opportunities. Mentorship was included in several strategies.

#### Setting targets and monitoring performance

Several strategies described audit activities to gather ‘baseline’ research activity data against which targets for improvement could be set, thus developing a culture for continuous improvement. Strategies included provision for monitoring and reporting progress against these targets (INF). Most would monitor research activity performance by number of studies undertaken, levels of staff and PPI engagement in studies (CTP), and recruitment rates into studies. Targets for recruitment rates on portfolios studies were set externally by the NIHR CRN, and linked to income for the Trust (SUS). The number of grant applications, grant-funded or commercially sponsored research studies was often cited (SUS). Monitoring of research outputs (peer reviewed publications and conference presentation) was common (DIS).

Targets for research income were evident and most strategies included a level of desirable growth in income (SUS). Links with industry were evident in target setting (LINKS). One strategy referred to shared performance targets with commercial partners, and another aimed to assess which partnerships were better at achieving commercial income (SUS).

Most strategies set objectives around staff engagement in research activities. These included an increase in ‘research ready’ staff or research activity in named staff groups, and some specified a desirable number of people at stages within a clinical academic pathway. Planned mechanisms for achieving this included developing research skills in staff (SKILLS) and links to academic networks and universities (LINKS).

Plans to provide regular performance reports to the Board of Directors were also included in a number of strategies, reflecting high level ‘hard-wiring’ into the organisation (INF). One strategy described a structure of distributed leadership ‘across the organisation from the Trust Board, through care groups into directorate and into clinical teams’ (CTP). It was thought that these leaders could enable enthusiasm for research, and spread innovation from research active groups to other parts of the organisation (DISS).

#### Internal investment: allocating resources to promote research capacity

The strategies highlighted the existing and planned use of organisational and financial resource to support RCD (SUS). The financial resource for this purpose came from ‘invest to save’ strategic use of trust finance, and from research income. The latter included grants, commercial collaborations, NIHR portfolio study recruitment funds, NIHR ‘between grant’ research capacity funds, and charitable monies linked to the organisation. An activity strongly aligned to supporting a research culture was through executive level support to identify additional resources agreed through ‘matched’ funding into research collaborations such as the CLAHRC YH. ‘Matched funding’ is a process by which members of the NHS workforce work with research teams to undertake research and implementation projects that align to the trust objectives. The NHS organisation agrees to provide practitioner and manager time into the project, and the academic’s time is externally funded and freely available to the trust. This reciprocal arrangement increases access to practical and methodological support.

How resources were utilised varied amongst the strategies. Many described a funding distribution model, agreed at a senior level to recompense research activity and incentivise clinical engagement in research priorities (CTP), as well as focusing on likely return on this investment (SUS).

Internal investment was planned to fund: research support services (e.g. research governance functions, PPI, and portfolio study recruitment activities at English sites) (INF); other training and support activities, typically involving funding academics to provide training and work with practitioners and managers to prepare grant applications (SKILLS); protected research time in practitioners’ job plans to ‘legitimise’ research alongside practice commitments (often as in recognition of previous research activity) (INF); joint clinical/academic posts with academic partners (LINKS); and seed-corn priority setting events and funds to develop research ideas with academics (LINKS).

## Conclusions

### Activities and RCD principles: making research ‘core business’ in health organisations.

We have described a range of activities identified through thematic analysis of ten NHS organisations’ R&D strategies in two countries in the UK. Whilst the data arises from planning documents in health services in high income countries, the activities address a range of principles (see Table 2) developed from a framework shaped by international evidence [13], indicating potential for building research capacity elsewhere. This paper offers some concrete examples of how the principles can be articulated in strategy documents. Targeted at organisational level, the activities described aim to make research ‘core business’ in a full range of healthcare organisations. We propose that these would be good candidate ideas for other health organisations planning RCD strategies.

The activities demonstrate a complex interplay between planning at an organisational level to develop a strong internal infrastructure, and undertakings that support individual career planning. They also aim to build stronger inter-organisational relationships and networks within health systems. The approach planned by many of the ACORN organisations is multi-layered and multifaceted, which has been shown to be effective elsewhere [8, 13, 23], and affirms observations made by others about the complexity of effective RCD organisational ‘interventions’ [14, 16, 24, 25].

### Strengths of activities for RCD

A number of activities cover the majority of RCD principles as presented in Table 2. These include: setting targets and monitoring performance; investing in internal resource for capacity development; developing and sustaining research collaborations; and developing research priorities, along with PPI. These activities balance inward looking and outward looking approaches, reinforcing the need for collaboration and networking recognised by others. Partnership development has been found to be an important determining factor for successful RCD across different organisations [24–26].

The only activity that addresses all RCD principles is that of setting targets and monitoring performance. This is an interesting observation: it is well recognised that measuring RCD is challenging. For example, Vasquez et al. [27] state that there is ‘limited consensus on and precedence for systematic evaluations of HRCS [health research capacity strengthening] initiatives, making it difficult to establish a clear benchmarks for success ‘, but it appears that the majority of these ACORN organisations have an ambition to do this. Some of this monitoring includes traditional measures, for example grant income and peer reviewed publications, whilst other ideas are more innovative, tracking practitioner, manager and service user engagement in research projects and planning. Levine et al. [25] advocate a concurrent use of different success criteria in order to capture process, and enhance cross-organisational comparison. The importance of such monitoring will bring shared learning across the ACORN group as this community of practice progresses.

From Table 2 it can be seen that the RCD principles are not equally represented by the core activities. The principle that is evident across the most activities is that of infrastructure development. This is unsurprising as this principle is very tangible at an organisational level, and it is easy to operationalise. Infrastructure development could also be considered first step in the R&D developmental of a health provider organisation. Levine et al. [25] have noted that infrastructure development is associated with the how established research activity is within an organisation, with novice ‘seed’ organisations having little or no research infrastructure and ‘fertilizer’ organisations having a well-developed research infrastructure. Healthcare provider organisations are more likely to be the former, and planning to establish and maintain an infrastructure is an important element of planning RCD at an organisational level. The strategy documents we examined have articulated important activities which can help achieve this.

The ‘close to practice’ principle is also evident within many activities, demonstrating an aspiration to involve practitioners, managers and patients throughout the research cycle. Many organisations realise this particular ‘offer’ by contributing to academic-practice partnerships, particularly in relation to PPI, and aim to monitor the impact of this activity through linked grant capture. However there was less measurement around clinical impact of research in services, although this was often a stated ambition of the strategy.

Much of the ‘hard wiring’ activity aims to make research ‘everybody’s business’ for example, within job descriptions, through mentoring, and integrating research aims in job plans, appraisal and performance management. There are also plans to reduce barriers to research engagement through establishing protected time for practitioners to do research and providing support for researchers to navigate the complex system of ethics and governance approvals. This approach is important as experiential ‘learning by doing’ is associated is in clinical academic career development [12, 28, 29] .

Authors have stressed the importance of providing funds for developing and sustaining research capacity [8, 30]. This is reinforced by our findings. Financial and business planning was woven throughout the strategy documents, underpinned by financial incentives for recruitment, making strategic judgements about return on investment, and assessment of partners who are likely to achieve commercial income or further grant capture. The national funding body (NIHR) had a great deal of influence in shaping the NHS business case. This included activity funded through recruitment incentives, in the use of ‘match funding’ in research partnerships like the CLAHRC, and by providing capacity funds associated with ‘between grants’ activity (RCF). It is noticeable that developing links with industry was evident across the strategies, which contradicts reports elsewhere in regard to RCD in NHS organisations [14], and could reflect more recent national NIHR policy and guidance. The aim of such business planning is to use resulting funds to strengthen the amount of capacity building activity and clinical academic careers. Whether this ambition is realised is yet to be established, however.

### Under-represented principles: opportunities for improving impact

Actionable dissemination is the least well represented principle (see Table 2). These findings are consistent with those of a qualitative study exploring barriers, motivators and critical success factors in establishing a strong research culture within AHPs in Australia [4]. Many of the trusts have planned to use traditional benchmarks of peer reviewed publications as a measure of research capacity, rather than outputs that could have an impact on clinical practice, and local dissemination was limited to newsletters and local conferences rather than using ‘actionable’ outputs. However, the use of such ‘boundary objects’ have been found to be useful ways of bridging the research-practice gap [11], and can demonstrate direct benefits to the clinical and quality objectives of an NHS organisation.

### Summary: how to make research ‘core business’ in health organisations

Many ACORN organisations aim to ‘hard wire’ RCB through strategically planning a range of RCD activities through linking research planning to wider organisational and business planning processes. The strategic plans were dominated by developing a strong infrastructure, and activities that enable research ‘close to practice’ were also apparent. Actionable dissemination was less evident in these plans: the model was primarily one of research production rather than research use and application. The strategic plans covered a broad range of target setting and monitoring performance, offering potential for measuring the impact of these plans on RCD, and for learning from one another in the ACORN group. The impact of research on clinical services and direct benefit for patients was missing, and warrants further investigation.

Our findings have demonstrated how research funders can influence health systems and capacity building through providing incentives for research activity, and supporting creative funding matched arrangements, where practitioner and health managers’ time is matched with grant funds to cement health systems engagement in such collaborations. ‘Between grant funding’ also supports organisational planning to sustain and strengthen capacity.

This paper offers some examples of how a number of health organisations are planning to build and sustain research capacity, and measure progress, and offers some ideas for further examination and debate. Our findings are based on written plans, which are recognised as an important step in organisational change [31]. The implementation and impact of such plans are not described, but would be worthy of investigation. Nevertheless our findings offer some interesting insights into how national policy and research funders can influence the plans of health systems engagement in research.

## Additional file

Additional file 1: Final coding tree for the activity codes. (DOCX 19 kb)

## Abbreviations

(L)CRN: (Local) Clinical Research Network.
ACORN: Addressing Capacity in Organisations to do Research Network.
AHP: Allied Health Professional.
CBU: Clinical Business Unit.
CLAHRC: Collaboration for Leadership in Applied Health Research and Care.
CTP: Close to Practice (from the Cooke framework).
DISS: Actionable Dissemination (from the Cooke framework).
INF: Infrastructure (from the Cooke framework).
LINKS: Linkages and Collaborations (from the Cooke framework).
NHS: National Health Service.
NIHR: National Institute for Health Research.
PPI: Patient and Public Involvement.
R&D: Research and Development.
RCB: Research Capacity Building.
RCD: Research Capacity Development.
RCF: Research Capability Funding.
RDS: Research Design Service.
REF: Research Excellence Framework.
SKILLS: Skills Development (from the Cooke framework).
SUST: Sustainability (from the Cooke framework).
