# Supplementary material for: How do NHS organisations plan research capacity development? Strategies, strengths, and opportunities for improvement
Source: BMC Health Serv Res. 2018 Mar 22;18:198. doi: 10.1186/s12913-018-2992-2 (PMC5865402; doi:10.1186/s12913-018-2992-2)
Supplement: Supplementary file 1 — Final coding tree for the activity codes. (DOCX 19 kb) [file 12913_2018_2992_MOESM1_ESM.docx]

| **Audit activities** |
| --- |
| Audit (research) skills and training received |
| Audit need for enhanced infrastructure |
| Audit of research activity and awareness |
| Audit protected research time |
| Audit researcher involvement in local, national, international research and policy forums |
| Clinical audit |
| Monitoring and reporting research activity, outputs, impact, and performance |
| REF submissions |
|  |
| **Organisational integration** |
| * Developing research priorities |
| Hard wired into the organisation: making research core business |
| IP commercialisation and exploitation |
| Leadership |
| Recruitment and retention of research-active staff |
| Research activity is Linked to staff award (reward) mechanism |
| Research Centre or Unit designation |
| Setting targets and monitoring performance |
| Staff management |
|  |
| **Partnerships** |
| Developing and sustaining research collaborations |
| Participate in research networks and policy forums |
| Patient and public involvement and engagement with research |
| Post creation (includes joint academic-clinical posts) |
|  |
| **Research dissemination and implementation** |
| Academic dissemination |
| Evidence based practice and knowledge transfer |
| Internal dissemination |
| Marketing and engagement activities |
| Website activities |
|  |
| **Research funding and sustainability** |
| External research funding |
| External investment in research (e.g. match funding, commissioning research) |
| Internal investment: allocating resources to promote research capacity |
|  |
| **Supporting research activities** |
| Financial management (including costings) |
| Generic support for research and innovation etc. |
| Information resources |
| Internal support networks and research interest groups |
| Proactive and timely communication of research opportunities |
| Project (study) management and co-ordination, quality processes etc. |
| Research governance support |
| Statistical or other methodological expertise (e.g. data analysis) |
| Study recruitment support |
|  |
| **Targets and research activity** |
| Conduct research (where not captured by other codes) |
| Patient participation targets |
| Student projects |
| Study approval time targets |
|  |
| **Training and development** |
| Academic study |
| Ethics, research governance, and Good Clinical Practice (GCP) |
| Induction of new staff |
| Research awareness training |
| Research skills development |
| Sources of support (e.g. statistics) and available training |
| Training for patients, carers, and public |
| Training needs analysis |
